# Association of online activities with obstetrics and gynecology specialty choice: a nationwide online survey

**DOI:** 10.5116/ijme.6320.36eb

**Published:** 2022-09-28

**Authors:** Yuto Maeda, Akihiro Hasegawa, Ryuta Miyake, Mihoko Dofutsu, Yayoi Higuchi, Daiken Osaku, Tokumasa Suemitsu, Yohei Onodera, Makio Shozu, Kiyonori Miura, Yoshio Yoshida, Hiroaki Komatsu, Hidemichi Watari

**Affiliations:** 1 Department of Global Health Promotion, Tokyo Medical and Dental University, Tokyo, Japan; 2 Department of Obstetrics and Gynecology, The Jikei University School of Medicine, Tokyo, Japan; 3 Department of Obstetrics and Gynecology, Nara Medical University, Japan; 4 Department of Obstetrics and Gynecology, Yokohama City University School of Medicine, Yokohama, Japan; 5 Department of Obstetrics and Gynecology, Kochi University, Kochi, Japan; 6 Department of Obstetrics and Gynecology, Tottori University, Tottori, Japan; 7 Department of Obstetrics and Gynecology, Kameda Medical Center, Chiba, Japan; 8 Department of Obstetrics and Gynecology, Akita University Graduate School of Medicine, Akita, Japan; 9 Evolution and Reproductive Biology, Medical Mycology Research Center, Chiba University, Chiba, Japan; 10 Department of Obstetrics and Gynecology, Nagasaki University Graduate School of Biomedical Sciences, Nagasaki, Japan; 11 Department of Obstetrics and Gynecology, University of Fukui, Fukui, Japan; 12 Department of Obstetrics and Gynecology, Hokkaido University Graduate School of Medicine, Sapporo, Japan

**Keywords:** Obstetrics and gynecology, online activities, face-to-face activities, COVID-19

## Abstract

**Objectives:**

To investigate the association between online activities and the number of new obstetrics and gynecology senior residents.

**Methods:**

A nationwide web-based, self-administered anonymous survey was conducted to investigate recruitment and clerkship activities during the coronavirus disease 2019 pandemic. An online questionnaire was sent to 576 obstetrics and gynecology training institutions in Japan between December 21, 2020, and January 31, 2021. Overall, 334 institutions that gave valid responses were included (response rate: 58.0%). Multivariate logistic regression analysis examined the association between online activities, including recruitment and clerkship activities, and the number of new obstetrics and gynecology senior residents in 2021. The stratified analysis by implementing face-to-face activities was conducted to clarify the association.

**Results:**

The number of new senior residents increased in 187 facilities (56.0%) and decreased in 147 facilities (44.0%). The facilities that implemented face-to-face and online activities were 185 (55.4%) and 120 (35.9%), respectively. In multivariate logistic regression analysis, an increased number of new obstetrics and gynecology senior residents was significantly associated with face-to-face activities (adjusted odds ratio (AOR)=2.58, 95% confidence interval (CI): 1.11–5.97, p<.001) but not with online activities. In the stratified analysis, online activities were significantly associated with an increased number of new obstetrics and gynecology senior residents among the facilities without face-to-face activities (AOR=3.81, 95% CI: 1.40–10.32, p=.009) but not among those with face-to-face activities (AOR=0.87, 95% CI: 0.42–1.78).

**Conclusions:**

Online activities were associated with an increased number of new obstetrics and gynecology senior residents among the facilities that did not conduct face-to-face activities.

## Introduction

Sexual and Reproductive Health and Rights (SRHR) for women are incorporated into the Sustainable Development Goals and are important for peace and prosperity in all nations. To achieve SRHR, obstetricians and gynecologists play a crucial role; thus, ensuring an increased number of new obstetricians and gynecologists is a global issue.

Choosing a specialty is an important decision for medical doctors. Most medical students are interested in deciding on a specialty when they enter university, but some develop the interest through clerkships or working as junior residents.^1^^,^^2^ As promoting factors for choosing Obstetrics and Gynecology (OB-GYN) as a specialty, previous studies showed that clerkship experience was important.^1^^,^^2^ This could be explained by the fact that clerkship experiences could provide opportunities for surgery, various clinical experiences, interaction with OB-GYN senior residents or other applicants, and fast-paced and acute experiences, attracting applicants to OB-GYN.^1^^,^^3^ However, severe acute respiratory syndrome coronavirus-2 infection has completely disrupted the traditional face-to-face clinical practice and the standard process of intern work, thus preventing interactions with senior residents and other applicants.^4^^-^^6^ Student participation in clinical practice has become unfeasible nationwide to prevent the spread of infection from patients to students and vice versa and to limit personal protective equipment use. Since most of the clerkships in obstetrics and gynecology involve procedures that require face-to-face interaction, such as deliveries and surgeries, medical students or junior residents from this field are vulnerable to the loss of clinical practice opportunities due to the COVID-19 pandemic in terms of conveying the appeal of the specialty.

Online education is one of the methods utilized to counteract the lack of real interaction caused by the COVID-19 pandemic. A survey of 3348 medical students in Libya regarding the use of e-learning six months after the COVID-19 pandemic began showed that 65% (n = 2176) of the students participated in online study groups and discussions, and 54.1% (n = 1811) reported that two-way communication was possible online.^7^ Previous studies have reported the benefits of online education and the ambivalent attitudes of students toward online education in OB-GYN.^8^^,^^9^ A cross-sectional study of 121 students in Germany showed high satisfaction with e-learning in the OB-GYN program consisting of online lecture notes, video materials, and online webinars (median score: 3.6-3.9 using a 5-point Likert scale).^8^ Another survey conducted following an online course on 98 students in Germany reported that online education programs, including online lectures, video tutorials based on real patients, and digital teaching on practical gynecological skills and examinations, achieved ratings as "good" or "excellent" among > 80% of the students. In contrast, 74% of the students desired bedside learning with real patients.^9^

Using online tools may positively impact specialty selection because they enable medical students or junior residents to experience the appeal of OB-GYN and to collect information on the specialty. However, no studies have focused on the association of online activities with specialty selection during the COVID-19 pandemic. In this study, we conducted a nationwide hospital-based survey to determine the association between online activities and the number of new senior residents majoring in OB-GYN.

## Methods

### Study design and Participants

We used the data from a nationwide web-based, self-administered anonymous survey to investigate the recruitment activities under the COVID-19 pandemic conducted by The Japanese Society of Obstetrics and Gynecology (JSOG) between December 21, 2020, and January 31, 2021. The questionnaire was provided to all 576 obstetrics and gynecology training facilities from the JSOG as online participants, and a letter was sent to the training directors of the facilities as a reminder. These facilities ranged from urban perinatal centers to regional obstetric care facilities and covered eastern and western parts of Japan. Of 576 participating facilities, 334 facilities (response rate: 58.0%) that sent valid responses were included in this study. Completion of the web-based questionnaires implied informed consent. All the data were collected anonymously in that survey, and no correspondence table exists. Since this study used data that had already been unlinked and anonymized prior to the study, and informed consent was obtained upon completion of the web-based questionnaire, Ethics Review Board approval was not required.

### Measurement

The primary interest outcome, the number of new OB-GYN senior residents, was asked by using the following question: "How did the number of people who decided to come to your hospital for obstetrics and gynecology training change this year compared with previous years? " Please choose the opinion that most closely matches your own: include no one, under 0.5 times, 0.5 to less than 1.0 times, same as average years, 1.0 to less than 1.5 times, 1.5 to less than 2.0 times, and more than twice. The reasons for asking the percentage compared with previous years rather than the number of new OB-GYN residents were as follows: some of the participating OB-GYN training facilities have many residents yearly. In contrast, others admit almost no new residents. We asked each facility for the percentage of new residents compared with that in previous years rather than the exact number to clarify the association between online activities and the number of new OB-GYN applicants during the COVID-19 pandemic. We defined a >1.0-fold increase as an increase and <1.0-fold increase as a decrease.

The primary exposure was online activities, including some recruitment and clerkship activities, which could affect the specialty choices of junior residents, such as information sessions, hospital tours, interviews, hands-on seminars, convivial parties, lectures, and clinical practice in inpatient or outpatient settings, such as physical or pelvic examination, ultrasound, surgery, and surgical training (e.g., ligation, suture, or dry box training for laparoscopic surgery).^2^^,^^3^^,^^10^ The content validity of recruitment and clerkship activities was reviewed when the questionnaire was made by obstetricians and gynecologists in charge of recruitment in Japan. Implementation of online activities was asked by using the following question: "Regarding recruitment activities and clinical practice at your hospital, please select the status of implementation after the COVID-19 pandemic (multiple selections are possible): never implemented, cessation after the pandemic, implemented face-to-face after the pandemic, implemented online after the pandemic, and implemented in other ways after the pandemic." We defined implementation of online activities as positive if participants selected "implemented online after the pandemic" for any one or more of the items.

As covariates, data on the location of the hospital, hospital status (i.e., university hospital or general hospital), the number of full-time obstetricians and gynecologists, and implementation of face-to-face activities were collected using an online questionnaire. The implementation of face-to-face activities was assessed using the same question for the implementation of online activities described above.

In addition, to examine the effect of the pandemic on recruitment activity, the following questions were asked: "Do you think the COVID-19 pandemic has affected the way you recruit obstetricians and gynecologists? please select each of the following: not at all, not significantly, partially, and significantly." "To what extent you were able to convey the appeal of obstetrics and gynecology to students and residents rotating at your hospital this year compared to previous years? Please indicate this on a scale of 0–10,"

### Statistical analysis

The chi-square test and Student's t-test were used to examine discrete and continuous variables, respectively. They compared background characteristics stratified by the number of new obstetrics and gynecology senior residents in that year compared with previous years. We then performed simple and multiple logistic regression analyses to examine the association between online activities and the number of new obstetrics and gynecology senior residents. In these analyses, two models were utilized. In model 1, we considered the institution's area, type, and number of full-time obstetricians and gynecologists in each facility. This was because these factors can affect lifestyle,^2^ stress levels,^11^ and the time demands of specialty work,^10^ which were reported as key factors influencing the application for obstetrics and gynecology residencies.^12^ Regarding institution areas, 47 prefectures in Japan were categorized into 10 areas commonly used in Japan (i.e., Hokkaido, Tohoku, Kanto, Tokyo, Hokuriku, Chubu, Kinki, Chugoku, Shikoku, Kyusyu, and Okinawa). The number of full-time obstetricians and gynecologists was categorized in increments of 5.

In model 2, we considered the variables used in model 1 and the implementation of face-to-face activities as covariates because face-to-face activities such as clerkships and hands-on seminars are well-known factors that positively affect the increased number of applicants for obstetrics and gynecology residencies.^13^^-^^15^ To clarify the association between online activities and the number of new OB-GYN senior residents considering face-to-face activities, we examined the interaction effect of online and face-to-face activities. We conducted stratified analyses according to the implementation of face-to-face activities. Statistical analyses were performed using Stata SE 15 (STATA Corp, College Station, TX, USA), and p<.05 was considered a statistically significant difference.

## Results

The background characteristics of the facilities and results of the questionnaire are shown in Table 1. The number of new OB-GYN senior residents increased in 187 facilities (56.0%) (defined as the increasing group). It decreased in 147 facilities (44.0%) (defined as the decrease group) in 2021, compared to the number in previous years. The proportions of facilities that implemented face-to-face and online activities were significantly higher in the increase group than in the decrease group (65.78% (n=123) vs. 42.18% (n=62), χ^2^_(1,N=334)_=18.55, p<0.01; 41.71% (n=78) vs. 28.57% (n=42), χ^2^_(1,N=334)_=6.17, p=.01, respectively). The number of OB-GYN staff in the facilities tended to be higher in the increasing group than in the decreasing group, but the difference was insignificant (χ^2^_(6, N=334)_ =11.14). The achievement rate for conveying the appeal of obstetrics and gynecology compared with that of previous years was higher in the increasing group (M=4.24, SD=1.68) than in the decreasing group (M=3.50, SD=1.49), (t_(__332)_=-4.22, p<.01). No other characteristics differed between the two groups. The association between the increase in new obstetrics and gynecology senior residents and online activities or covariates is presented in Table 2. Online activities were significantly associated with an increase in the number of new obstetrics and gynecology senior residents in the crude analysis and model 1 (adjusted odds ratio [AOR]=1.94, [95% confidence interval [CI]]: 1.15–3.26, p=.01). However, this association was not found in model 2 when the implementation of face-to-face activities was adopted as a covariate. The implementation of face-to-face activities was significantly associated with an increase in the number of new obstetrics and gynecology senior residents (AOR=2.67, 95% CI: 1.60–4.44, p<.01). Hospitals with 11-15 or 16-20 full-time obstetricians and gynecologists significantly had new OB-GYN senior residents compared with those with 1-5 full-time obstetricians and gynecologists (AOR=3.82, 95%CI: 1.69–8.66, p<.01, and AOR=3.09, 95%CI: 1.13–8.43, p=.03, respectively).

**Table 1:** Background characteristics stratified by the change in the number of senior residents in 2021

Activity	Facilities with increase in residents in 2021 (n=187)	Facilities with decrease in residents in 2021 (n=147)	p-value
	N (%)	N (%)	
Face-to-face activity^*^	123 (65.78)	62 (42.18)	<0.01
Online activity^*^	78 (41.71)	42 (28.57)	0.01
Type of facilities			0.98
University Hospital	60 (32.09)	47 (31.97)	
Others^†^	127 (67.91)	100 (68.03)	
Number of obstetricians and gynecologists			0.08
1-5	35 (18.72)	48 (32.65)	
6-10	64 (34.22)	48 (32.65)	
11-15	39 (20.86)	19 (12.93)	
16-20	27 (14.44)	14 (9.52)	
21-25	10 (5.35)	8 (5.44)	
26-30	4 (2.14)	3 (2.04)	
31 or more	8 (4.28)	7 (4.76)	
Location of the institutions			0.61
Hokkaido	4 (2.14)	7 (4.76)	
Tohoku	12 (6.42)	10 (6.80)	
Kanto	40 (21.39)	33 (22.45)	
Tokyo	20 (10.70)	21 (14.29)	
Hokuriku	13 (6.95)	5 (3.40)	
Chubu	16 (8.56)	12 (8.16)	
Kinki	37 (19.79)	25 (17.01)	
Chugoku	16 (8.56)	7 (4.76)	
Shikoku	8 (4.28)	9 (6.12)	
Kyusyu/Okinawa	21 (11.23)	18 (12.24)	
Feeling of the success in conveying the appeal of obstetrics and gynecology compared with the average year^‡ ^**Mean (SD)**	4.24 (1.68)	3.50 (1.49)	<0.01
Feeling of the influence on recruit activity by the COVID-19 pandemic			0.93
not at all	6 (3.21)	5 (3.40)	
not so much	56 (29.95)	41 (27.89)	
partially	76 (40.64)	65 (44.22)	
strongly	49 (26.20)	36 (24.49)	

*Face-to-face or online activities included lectures, information sessions on facilities, hospital tours, interviews, hands-on seminars, convivial parties, and clinical practice on inpatient or outpatient activities; †City hospital, a medical clinic with bed; ‡10-point scale, 5-point scale: same to average years

**Table 2:** Association between an increase in the number of senior residents in 2021 and online activities or covariates

Activity and facilities	Crude OR [95% CI]	Model 1 (OR [95% CI])^ *^	Model 2 (OR [95% CI])^ †^	p-value
Implementation of online activities	1.79 (1.13-2.84)	1.94 (1.15-3.26)	1.66 (0.97-2.84)	0.07
Implementation face-to-face activities	2.63 (1.69-4.11)	-	2.67 (1.60-4.44)	<0.01
Type of facilities				
Others	reference	reference	reference	
University hospital	0.99 (0.62-1.58)	1.89 (0.92-3.89)	2.12 (1.00-4.48)	<0.01
Number of full-time obstetricians and gynecologists				
1-5	reference	reference	reference	
6-10	1.83 (1.03-3.25)	1.98 (1.08-3.66)	1.81 (0.96-3.41)	0.07
11-15	2.82 (1.40-5.67)	3.67 (1.65-8.16)	3.82 (1.69-8.66)	<0.01
16-20	2.64 (1.21-5.76)	4.04 (1.53-10.67)	3.09 (1.13-8.43)	0.03
21-25	1.71 (0.61-4.79)	2.85 (0.80-10.15)	2.18 (0.59-8.03)	0.24
26-30	1.83 (0.38-8.69)	3.41 (0.60-19.40)	2.88 (0.49-16.99)	0.24
more than 30	1.57 (0.52-4.73)	2.16 (0.54-8.61)	1.57 (0.38-6.45)	0.53
Location of the institutions				
Tokyo	reference	reference	reference	
Hokkaido	0.60 (0.15-2.37)	0.94 (0.22-4.04)	0.91 (0.20-4.09)	0.91
Tohoku	1.26 (0.45-3.56)	1.76 (0.57-5.40)	1.29 (0.41-4.06)	0.66
Kanto	1.27 (0.59-2.74)	1.55 (0.69-3.52)	1.53 (0.66-3.52)	0.32
Hokuriku	2.73 (0.82-9.06)	3.69 (1.04-13.15)	3.35 (0.92-12.2)	0.07
Chubu	1.40 (0.53-3.68)	1.31 (0.47-3.69)	1.45 (0.51-4.16)	0.49
Kinki	1.55 (0.70-3.44)	1.87 (0.79-4.40)	1.73 (0.72-4.18)	0.22
Chugoku	2.40 (0.82-7.06)	3.35 (1.07-10.52)	3.09 (0.97-9.87)	0.06
Shikoku	0.93 (0.30-2.90)	1.54 (0.46-5.16)	1.65 (0.47-5.76)	0.43
Kyusyu/Okinawa	1.22 (0.51-2.95)	1.66 (0.65-4.22)	1.40 (0.54-3.63)	0.49
Interaction effect				
Online activity× Face-to-face activity			0.26 (0.09-0.76)	0.01

*Model 1: adjusted by type of facilities, number of obstetricians and gynecologists, and pandemic area †Model 2: adjusted by covariates used in model 1 and face-to-face activities

The interaction effect of the implementation of online and face-to-face activities was observed (AOR=0.26, 95%CI: 0.09–0.76, p for interaction=.01). Other covariates were not associated with such an increase. In the stratified analysis, the implementation of online activities was significantly associated with an increase in the number of new OB-GYN senior residents among the facilities that did not conduct face-to-face activities (AOR=3.81, 95% CI: 1.40–10.32, p=.01) but not among those that conducted face-to-face activities (Table 3).

**Table 3:** Association between an increase in the number of senior residents in 2021 and online activities stratified by the implementation of face-to-face activities

Activities	n (%)	Adjusted OR [95% CI]^ *^	p-value
Face-to-face activities (-)			
Online activities (-)	115 (77.1)	reference	
Online activities (+)	34 (22.82)	3.81 (1.40-10.32)	0.01
Face-to-face activities (+)			
Online activities (-)	99 (53.51)	reference	
Online activities (+)	86 (46.49)	0.87 (0.42-1.78)	0.70

*Covariates: type of facilities, number of obstetricians and gynecologists, pandemic area

## Discussion

To the best of our knowledge, this is the first study to investigate the association between online activities and an increase in the number of new obstetrics and gynecology senior residents. Our multiple logistic regression analysis revealed a significant association between the increase in the number of new obstetrics and gynecology senior residents and online activities after adjusting for the institution's location, type, and the number of full-time obstetricians and gynecologists as covariates. However, this association was not found when the implementation of face-to-face activities was adopted as a covariate. The interaction effect and the stratified analysis indicated that online activities were significantly associated with an increase in the number of new obstetrics and gynecology senior residents among the facilities that did not conduct face-to-face activities.

The background characteristics demonstrated that 67.7% (n=226) of the total respondents partially or strongly felt the influence of COVID-19 on recruitment activity. In addition, even facilities with more senior residents in 2021 reported being less capable of conveying their appeal than in previous years. In Japan, clinical training in a hospital begins in most universities' fourth or fifth year of medical school. After six years of medical school and two years of internship after graduation, students begin their specialty training. They select a specialty based on their lifestyle preferences and interest throughout their clerkships and internships.^16^^,^^17^ Therefore, discontinuing clerkships and internships due to COVID-19 may have impacted the recruitment of obstetrics and gynecology applicants.

Online activity was significantly associated with an increase in the number of new obstetrics and gynecology senior residents in the facilities that did not conduct face-to-face activities. However, no association was found in facilities that conducted face-to-face activities. The possible explanation for this result is that face-to-face and online activities positively impacted the recruitment of obstetrics and gynecology senior residents by familiarizing them with obstetrics and gynecology and permitting interaction with senior residents. It has been reported that interaction with senior residents and the provision of information sessions are important in selecting a major.^18^ A study of 238 medical students in the United States regarding their decision to major in urology during the COVID-19 pandemic showed that several students considered one-on-one or small-group interactions with senior residents (83%, n=197) and learning about the facilities offering programs (72%, n=171) as very important when selecting a urology program.^18^ Further studies to examine the mechanism of online activities promoting the motivation for being obstetricians and gynecologists among students or interns are needed.

The strength of this study lies in the fact that it was a nationwide survey conducted within a limited number of facilities in Japan where senior residents can start major training. However, there were some limitations to this study. First, we essentially evaluated the activities of students undergoing clerkships and junior residencies. Given that the decision on a field of major can be influenced by multiple factors and is not made at a certain time, unmeasured factors could have affected the results. Second, since there was no golden standard for the categorization of online activities, we could not examine which type of activities especially had a positive impact on recruiting new OB-GYN senior residents. Third, due to the study design using a self-assessed questionnaire, the study was susceptible to some biases, such as recall, survival, and social desirability. Facilities that did not respond to the questionnaires might not be willing to answer the questionnaire, and those that responded to the questionnaire could report a higher resident number than they had, which may have affected the results. Finally, the increase in new obstetrics and gynecology senior residents was expressed as a percentage of the intake from previous years; thus, we could not evaluate the specific number.

In conclusion, online activities were associated with an increase in the number of new obstetrics and gynecology senior residents in the facilities that did not conduct face-to-face activities. Further studies are warranted to clarify whether face-to-face or online activities are superior, the effect of the combined use of both activities, and the types of online activities that are effective for recruitment.

### Acknowledgments

We are deeply grateful to all the facilities that responded to this survey. Also, we are deeply grateful to statistical experts Masashi Kizuki and Yuiko Nagamine for their statistical advice.

### Conflict of Interest

The authors declare that they have no conflict of interest.
